# Correlations of circulating miR-26b level with left ventricular hypertrophy and cardiac function in elderly patients with hypertension

**DOI:** 10.12669/pjms.37.4.4048

**Published:** 2021

**Authors:** Jian Fu, Fang Lin, Zhengxia Pan, Chun Wu

**Affiliations:** 1 Jian Fu, Department of Cardiac Surgery, Children’s Hospital of Chongqing Medical University, National International Science and Technology Cooperation Base for Children’s Developmental Diseases, Ministry of Education Key Laboratory of Child Development and Disorders, Chongqing Key Laboratory of Pediatrics, Chongqing 400014, P.R. China; 2 Fang Lin, The Second Affiliated Hospital of The Army Medical University, No. 136, Zhongshan Second Road, Yuzhong District, Chongqing 400014, P.R. China; 3 Zhengxia Pan, Department of Cardiac Surgery, Children’s Hospital of Chongqing Medical University, National International Science and Technology Cooperation Base for Children’s Developmental Diseases, Ministry of Education Key Laboratory of Child Development and Disorders, Chongqing Key Laboratory of Pediatrics, Chongqing 400014, P.R. China; 4 Chun Wu, Department of Cardiac Surgery, Children’s Hospital of Chongqing Medical University, National International Science and Technology Cooperation Base for Children’s Developmental Diseases, Ministry of Education Key Laboratory of Child Development and Disorders, Chongqing Key Laboratory of Pediatrics, Chongqing 400014, P.R. China

**Keywords:** miR-26b, Elderly, Hypertension, Left ventricular hypertrophy, Cardiac function

## Abstract

**Objectives:**

To study the correlations of circulating miR-26b level with left ventricular hypertrophy (LVH) and cardiac function in elderly patients with hypertension.

**Methods:**

A total of 132 eligible patients were divided into low and high miR-26b level groups. Their baseline clinical data and biochemical indices were compared. The correlations between miR-26b level and echocardiographic parameters were studied by Pearson’s analysis. Factors affecting LVH were explored by multivariate logistic regression analysis. The role of miR-26b in diagnosing LVH was predicted by receiver operating characteristic curve.

**Results:**

The relative expression level of miR-26b was 4.56-16.93, with a median of 7.62. The two groups had similar baseline clinical data and biochemical indices (P>0.05). Compared with high miR-26b level group, interventricular septal thickness (IVST), left ventricular posterior wall thickness (LVPWT), left ventricular mass index (LVMI) and number of LVH cases in low miR-26b level group significantly increased (P<0.05), and mitral ratio of peak early to late diastolic filling velocity (E/A) decreased (P<0.05). Circulating miR-26b level was negatively correlated with IVST, LVPWT and LVMI (P<0.0001), and positively correlated with E/A (P<0.0001). The proportion of cardiac hypofunction cases in low miR-26b level group significantly exceeded that of high miR-26b level group (P<0.05). Age and increased IVST, LVPWT and LVMI were independent risk factors for LVH (P<0.05), and elevated miR-26b level was a protective factor (P<0.05). AUC was 0.836, and the optimal cutoff value was 8.83, with high sensitivity and specificity.

**Conclusions:**

MiR-26b level is negatively correlated with LVH and positively correlated with left ventricular diastolic function in elderly hypertensive patients. It is a protective factor for LVH complicated with diastolic dysfunction and a potential biomarker for diagnosis.

## INTRODUCTION

As a common chronic disease bringing a serious economic burden, hypertension is mainly typified by the continuous rise of arterial blood pressure, and its long course changes the cardiac structure and function. The early manifestations of hypertension include left ventricular hypertrophy (LVH) and diastolic dysfunction. As the disease progresses, ventricular systolic dysfunction and enlargement of the heart cavity occur, eventually triggering heart failure.^1^ LVH is a common type of hypertension-induced target organ damage, mainly manifested as increased myocardial fibrosis. At present, its pathophysiological mechanism has not been fully clarified, but miRNAs have been verified to participate in cardiovascular pathophysiological processes such as heart development and remodeling, arrhythmia and myocardial hypertrophy.^2^ Circulating miRNAs, i.e. miRNAs in serum and plasma, can maintain extremely high stability under harsh conditions such as high temperature, acidity and alkalinity together with RNase .^3^ The correlations of changes in the circulating miR-26 level with LVH and cardiac function in elderly patients with hypertension have seldom been referred hitherto. In this study, therefore, the circulating miR-26b level and echocardiographic parameters in these patients were detected to explore their correlations, thereby providing some guidance for the early detection and diagnosis of LVH upon hypertension.

## METHODS

A total of 132 elderly patients with hypertension treated in our hospital from January 2017 to October 2019 were selected. They were aged 62-85 years old, including 80 males and 52 females. Hypertension was diagnosed based on the 2018 Chinese Guidelines for Prevention and Treatment of Hypertension^4^: The blood pressures of patients taking no antihypertensive drugs were measured three times in different days, with diastolic blood pressure (DBP) of ≥90 mmHg and/or systolic blood pressure (SBP) of ≥140 mmHg.

### Inclusion criteria

1) Patients aged over 60 years old; 2) patients who met the above diagnostic criteria for hypertension; 3) patients with primary hypertension; 4) patients who did not take antihypertensive drugs; 5) patients who signed the informed consent and actively cooperated in this study.

### Exclusion criteria

1) Patients with primary aldosteronism, pheochromocytoma, renal hypertension or other secondary hypertension; 2) patients with congenital cardiac malformation; 3) patients complicated with heart diseases such as hypertrophic cardiomyopathy, valvular heart disease and systolic heart failure; 4) patients with some diseases or those who had taken drugs affecting the heart rate; 5) patients with smoking, drinking or diabetes history; 6) patients complicated with severe liver, spleen, lung, kidney or other systemic diseases; 7) patients with metabolic diseases or tumors; 8) patients who received valve replacement, coronary artery bypass grafting, pacemaker implantation or surgery in the past year. This study was reviewed and approved by the Medical Ethics Committee of our hospital on January 6^th^ 2017.

### Baseline clinical data

The baseline clinical data of patients were collected, including gender, age, height and weight, and the body mass index (BMI) was calculated according to the following formula: BMI = weight (kg)/ [height (cm)]^2^. DBP and SBP of the patients measured by nurses in our hospital at admission were collected.

### Detection of circulating miR-26b level

The venous blood was collected using a disposable vacuum blood collection tube containing anticoagulant ethylenediaminetetraacetic acid 12 hour after fasting in the morning of the next day after admission. Then the venous blood was let still for 1-2 hour at normal temperature and centrifuged at 4°C to obtain the plasma that was transferred into a sterilized RNase-free tube. Subsequently, total RNA was extracted from the plasma by the TRIzol method and quantified using a spectrophotometer. Next, the 3’ end of miRNAs was treated with poly(A), and reverse transcription was carried out. Using the synthesized cDNA as the template, RT-PCR was performed by the SYBR Green I method. Primers for miR-26b: F: 5’-ACACTCCAGCTGGGTTTGGTCCCCTTCAAC-3’, R: 5’-GGTGTCGTGGAGTCGGCAATTCAGTTGAG-3’. Primers for U6: F: 5’-CTCGCTTCGGCAGCACA-3’, R: 5’-AACGCTTCACGAATTTGCGT-3’. The relative expression of miR-26b was calculated according to 2^-ΔCt^.

### Detection of biochemical indices

The venous blood was collected using a heparin sodium anticoagulation tube 12 hour after fasting in the morning of the next day after admission. Following centrifugation, the levels of fasting plasma glucose (FPG), total glyceride (TG), total cholesterol (TC), low-density lipoprotein cholesterol (LDL-C), high-density lipoprotein cholesterol (HDL-C), hemoglobin A1c (HbA1c), alanine aminotransferase (ALT), aspartate aminotransferase (AST), serum creatinine (Scr), creatine kinase (CK), creatine kinase-muscle/brain (CK-MB), lactate dehydrogenase (LDH), high-sensitivity C-reactive protein (hs-CRP), blood urea nitrogen (BUN) and uric acid (UA) were measured with Olympus AU400 automatic biochemical analyzer (Japan).

### Echocardiography

Echocardiography was performed by the same ultrasonographer. According to the criteria recommended by the American Society of Echocardiography^5^, the left ventricular end diastolic diameter (LVEDD), interventricular septum thickness (IVST), left ventricular posterior wall thickness (LVPWT), left ventricular ejection fraction (LVEF%), mitral valve early diastolic blood flow peak (E-Peak) and late diastolic flow peak (A-Peak) were measured by GE Vivid 7 color ultrasound system (USA) with a probe frequency of 2.5-3.5 MHz, and the ratio of E-Peak to A-Peak (E/A) was calculated. Three measured cardiac cycles were averaged. The left ventricular mass (LVM) was calculated based on the Devereux formula: LVM = 1.04 × [(LVEDD+IVST+LVPWT)^3^ - LVEDD^3^] - 13.6. The left ventricular mass index (LVMI) was calculated by the body surface area (BSA) according to the formula below: LVMI = LVM/BSA. LVMI of >125 g/m^2^ (male) and >110 g/m^2^ (female) indicated LVH.

### New York Heart Association (NYHA) classification

The patients were classified as per the standard formulated by NYH^6^: Class I: There is no limitation of physical activity, and ordinary physical activity does not cause fatigue, dyspnea, palpitation, shortness of breath and angina pectoris symptoms. Class II: There is slight limitation of physical activity, and ordinary physical activity results in fatigue, dyspnea, palpitation, shortness of breath or angina pectoris symptoms, but patients are comfortable at rest. Class III: There is marked limitation of physical activity, and light physical activity causes dyspnea, palpitation or shortness of breath, but patients are comfortable at rest. Class IV: Patients are unable to carry on any physical activity, and dyspnea, palpitation, shortness of breath, angina pectoris or cardiac insufficiency occurs at rest. Patients in class I had normal cardiac function, while those in class II-IV suffered from cardiac dysfunction.

### Statistical analysis

All data were statistically analyzed by SPSS 22.0 software. The numerical data were expressed as percentage [n (%)] and subjected to the χ^2^ test. The quantitative data were represented as mean ± standard deviation, and intergroup comparisons were conducted by the independent t test. The correlations between miR-26b level and various echocardiographic parameters were studied by the Pearson’s analysis. Multivariate logistic regression analysis was carried out to explore the factors affecting LVH. The role of circulating miR-26b in diagnosing LVH was predicted by plotting the receiver operating characteristic (ROC) curve. P<0.05 was considered statistically significant.

## RESULTS

### Baseline clinical data of patients with different miR-26b levels

The relative expression level of miR-26b in 132 patients was 4.56-16.93, with a median of 7.62, so patients with miR-26b levels of <7.62 and ≥7.62 were included into low and high miR-26b level groups, respectively. The two groups had similar baseline clinical data including gender, age, BMI, DBP and SBP as well as biochemical indices such as FPG, TG, TC, LDL-C, HDL-C, HbA1c, ALT, AST, Scr, CK, CK-MB, LDH, hs-CRP, BUN and UA (P>0.05) (Table-I).

**Table-I:** Baseline clinical data of patients with different miR-26b levels [case (%)] (*X̅*± s).

Item	Low miR-26b level group (n=76)	High miR-26b level group (n=56)	χ^2^/t	*P*
Gender (case)			1.217	0.269
Male	43	37		
Female	33	19		
Age (year)	71.85±7.03	72.16±7.09	0.249	0.803
BMI (kg/cm^2^)	25.41±3.28	24.92±3.37	0.838	0.403
DBP (mmHg)	93.52±8.24	92.87±8.15	0.450	0.653
SBP (mmHg)	148.93±13.14	150.12±13.78	0.504	0.615
FPG (mmol/L)	5.57±0.52	5.63±0.59	0.619	0.537
TG (mmol/L)	2.41±0.58	2.42±0.55	0.100	0.920
TC (mmol/L)	5.39±0.65	5.36±0.62	0.267	0.789
LDL-C (mmol/L)	2.62±0.31	2.59±0.28	0.572	0.568
HDL-C (mmol/L)	1.35±0.34	1.32±0.31	0.519	0.604
HBA1c (%)	5.18±0.67	5.24±0.69	0.502	0.616
ALT (U/L)	30.21±9.93	29.85±9.74	0.208	0.836
AST (U/L)	27.56±8.24	28.16±8.82	0.401	0.689
Scr (μmol/L)	75.94±13.79	77.01±14.35	0.433	0.666
CK (U/L)	96.48±22.56	95.39±21.78	0.278	0.781
CK-MB (U/L)	16.13±4.89	14.91±4.04	1.523	0.130
LDH (U/L)	144.76±31.25	141.28±30.97	0.635	0.527
HsCRP (mg/L)	12.37±5.68	11.94±5.26	0.443	0.658
BUN (mmol/L)	5.41±1.39	5.37±1.34	0.166	0.868
UA (μmol/L)	385.42±39.76	392.23±40.15	0.969	0.335

Compared with high miR-26b level group, IVST, LVPWT, LVMI and number of LVH cases in low miR-26b level group significantly increased (P<0.05), and the E/A ratio significantly decreased (P<0.05). The two groups had similar LVEDD and LVEF (P>0.05) (Table-II).

**Table-II:** Echocardiographic parameters of patients with different miR-26b levels [case (%)] (X̅ ± s).

Item	Low miR-26b level group (n=76)	High miR-26b level group (n=56)	χ^2^/t	*P*
LVEDD (mm)	44.57±3.94	44.25±3.86	0.465	0.643
IVST (mm)	12.23±1.17*	9.96±0.92	12.031	0.000
LVPWT (mm)	11.69±1.04*	9.57±0.95	12.003	0.000
LVEF (%)	65.72±6.48	66.13±6.54	0.358	0.721
E/A	0.59±0.14*	0.92±0.31	8.219	0.000
LVMI (g/m^2^)	139.35±14.25*	110.42±10.78	12.738	0.000
LVH (case, %)	70 (92.11)*	13 (23.21)	65.558	0.000

Compared with high miR-26b level group,

* P<0.05.

**Fig. 1:**
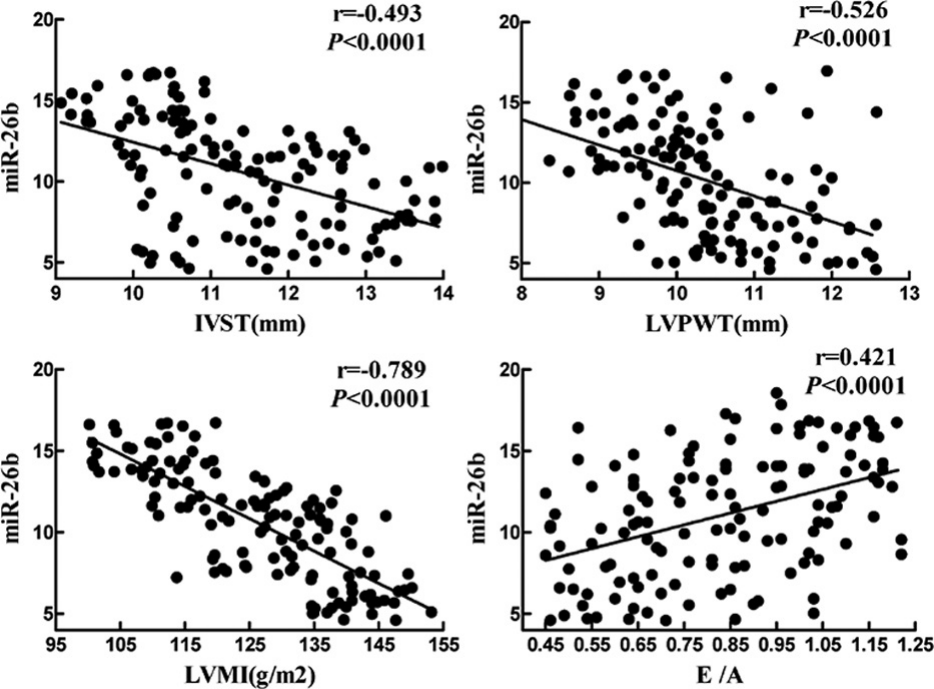
Correlations of miR-26b level with IVST, LVPWT, LVMI and E/A ratio.

The circulating miR-26b level was significantly negatively correlated with IVST, LVPWT and LVMI (r=-0.493, -0.526, -0.789, P<0.0001), and positively correlated with E/A (r=0.421, P<0.0001) (Fig.1).

The proportion of cardiac hypofunction cases in low miR-26b level group (72/76, 94.74%) was significantly exceeded that of high miR-26b level group (26/56, 46.43%) (P<0.05) (Table-III).

**Table-III:** NYHA classification of patients with different miR-26b levels [case (%)].

Item	Low miR-26b level group (n=76)	High miR-26b level group (n=56)	χ^2^	*P*
Class I (case, %)	4 (5.26)*	33 (58.93)		
Class II (case, %)	32 (42.11)*	19 (33.93)		
Class III (case, %)	30 (39.47)*	3 (5.36)		
Class IV (case, %)	10 (13.16)*	1 (1.79)		
Cardiac hypofunction (case, %)	72 (94.74)*	26 (46.43)	42.861	0.000

Compared with high miR-26b level group,

* P<0.05.

Age and increased IVST, LVPWT and LVMI were independent risk factors for LVH in elderly patients with hypertension (P<0.05), and elevated miR-26b level was a protective factor (P<0.05) (Table-IV).

**Table-IV:** Factors affecting LVH in elderly patients with hypertension.

Factor	β	SE	Wald	P	OR (95%CI)
Age	1.734	0.927	3.419	0.021	2.379 (1.368~3.842)
IVST	1.295	0.646	2.358	0.013	5.142 (3.405~6.257)
LVPWT	0.867	0.719	6.642	0.032	3.063 (2.241~5.463)
LVMI	2.453	0.882	4.734	0.006	4.581 (3.263~7.879)
miR-26b	-2.246	0.954	7.527	0.000	0.528 (0.257~0.895)

Age: >75 years old=1, ≤75 years old=0; IVST: increase=1, no increase=0; LVPWT: increase=1, no increase=0; LVMI: male>125 g/m^2^, female>110 g/m^2^=1, male≤125 g/m^2^, female≤110 g/m^2^=0; miR-26b: ≥7.62=1, <7.62=0.

**Fig. 2:**
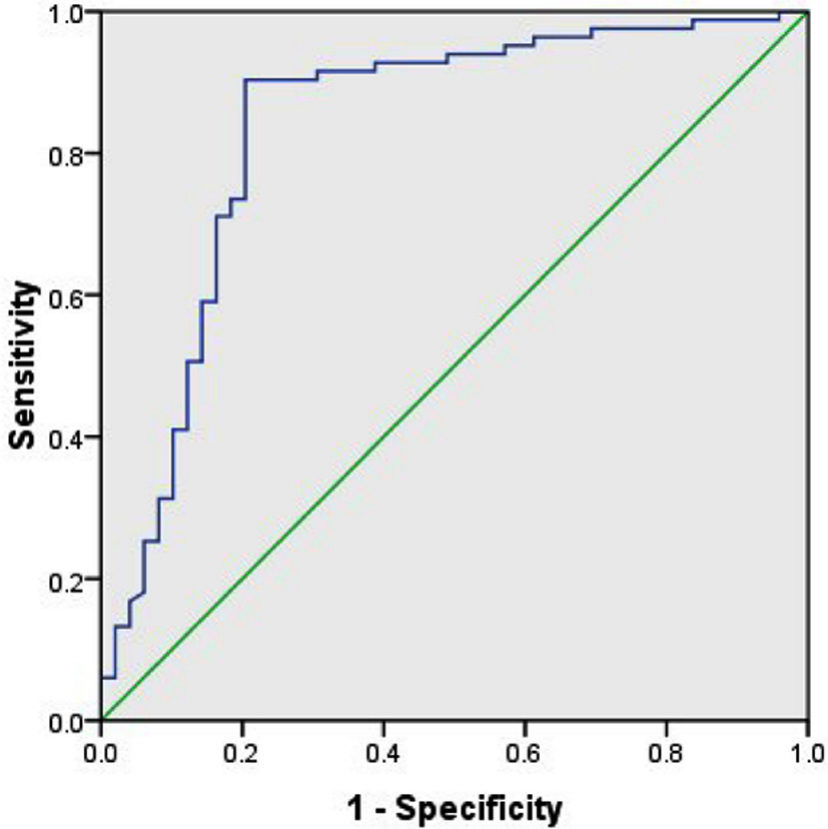
ROC curve of miR-26b level for LVH in elderly patients with hypertension.

The area under ROC curve was 0.836, with 95%CI of 0.757~0.916 (P<0.05). The optimal cutoff value was 8.83, the sensitivity was 81.4% and the specificity was 78.9%. Therefore, miR-26b level had high diagnostic value for LVH in elderly patients with hypertension (Fig.2).

## DISCUSSION

LVH is one of the common complications of hypertension. Long-term hypertension can increase the left ventricular afterload of the heart and thus leads to this chronic adaptive change, i.e. the occurrence of LVH.^7^ The rate of arrhythmia in patients with hypertension and LVH was significantly higher than that in patients with simple hypertension.^8^ The coronary reserve capacity of patients with hypertension and LVH is significantly reduced, resulting in acute myocardial ischemic events that easily occur and increase the risk of death.^9^ At present, the commonly used diagnostic methods such as electrocardiogram, magnetic resonance imaging and echocardiography have disadvantages or limitations.^10^ There is an urgent need to find a simple, specific and highly sensitive diagnostic method in clinical practice.

As endogenous non-coding small-molecule RNAs that are highly conserved during biological evolution, miRNAs can regulate gene expression at the post-transcriptional level.^11^ The roles of miRNAs in diseases such as viral diseases, digestive system, nervous system and reproductive system are well-documented.^12^ They have been involved in cardiac development and remodeling, and are closely related to various cardiovascular diseases such as myocardial fibrosis, myocardial hypertrophy and heart failure.^13^ The expression level of miR-26b in patients with coronary heart disease is significantly lower than that in healthy people, and miR-26b is expected to become a marker for the diagnosis of coronary heart disease.^14^ In a mouse model of atrial fibrosis and fibrillation, miR-26 inhibits the Ang II/KLF 4/TGF-β signaling pathway activation, thereby combating atrial fibrillation and atrial fibrosis.^15^ In this study, the miR-26b levels in the venous blood of 132 elderly hypertensive patients were detected. The results showed that the relative expression level was between 4.56 and 16.93, and the median was 7.62 as the group cut-off value, and the value of <7.62 was recorded as miR-26b low level group, ≥7.62 is recorded as miR-26b high level group. The two groups had similar baseline clinical data including gender, age, BMI, DBP and SBP as well as biochemical indices such as FPG, TG, TC, LDL-C, HDL-C, HbA1c, ALT, AST, Scr, CK, CK-MB, LDH, hs-CRP, BUN and UA (P>0.05).

Compared with high miR-26b level group, IVST, LVPWT, LVMI and number of LVH cases in low miR-26b level group significantly increased (P<0.05). IVST and LVPWT determine LVMI, and the left ventricular weight index can be directly used as an important clinical index to measure LVH. A high LVMI means more serious LVH.^16^ Thus, the lower the circulating miR-26b level, the more likely LVH occurs. Besides, an E/A ratio of <1 suggests decline of the left ventricular diastolic function.^17^ Elderly hypertensive patients with LVH often suffer from decreased left ventricular diastolic function.^18^ This study showed that the E/A ratio of both groups was <1, and the value of the miR-26b low level group was significantly lower than that of the miR-26b high level group (P<0.05). Accordingly, both groups of had left ventricular diastolic dysfunction, and the lower the miR-26b level, the more serious. Moreover, the circulating miR-26b level was significantly negatively correlated with IVST, LVPWT and LVMI (P<0.0001), and positively correlated with E/A (P<0.0001). The proportion of cardiac hypofunction cases in low miR-26b level group significantly exceeded that of high miR-26b level group (P<0.05). Collectively, elderly hypertensive patients with low miR-26b levels were more likely to have LVH and cardiac hypofunction.

Multivariate analysis showed that age and increased IVST, LVPWT and LVMI were independent risk factors for LVH in elderly patients with hypertension (P<0.05), and elevated miR-26b level was a protective factor (P<0.05). With increasing age, the volume of myocardial cells increases, the heart collagen component and heart weight increase, long-term overload pressure and left ventricular adaptive reaction failure may easily lead to LVH.^19^ The expression of miR-26a and miR-26b is down-regulated in rat cardiac hypertrophy tissues, and inhibiting the target glycogen synthase kinase-3β can regulate the cardiac hypertrophy process.^20^ Herein, miR-26b also played a regulatory role in the process of LVH in elderly hypertensive patients, but its mechanism remains elusive. ROC curve analysis showed that the area under the curve was 0.836, 95%CI was 0.757~0.916 (P<0.05), the optimal cutoff value was 8.83, the sensitivity was 81.4%, and the specificity was 78.9%, suggesting that the miR-26b expression level of <8.83 can be diagnosed as LVH sensitively and specifically.

## CONCLUSION

In summary, the circulating miR-26b level is negatively correlated with LVH and positively correlated with left ventricular diastolic function in elderly patients with hypertension. It is a protective factor for LVH complicated with diastolic dysfunction and a potential biomarker for the diagnosis of LVH in these patients.

### Authors’ contributions:

**JF & ZXP** Designed this study and significantly revised this manuscript.

**FL** Drafted this manuscript.

**FL & CW** Collected and analyzed clinical data.

All authors agree to the submission and publication of this manuscript.
